# Evaluation of the Translucency Properties for CAD/CAM Full Ceramic Crowns Fabricated From Glass Ceramics (E.max) or High Translucency Zirconia (Lava Plus): A Clinical Study

**DOI:** 10.7759/cureus.34935

**Published:** 2023-02-13

**Authors:** Chaza Kanout

**Affiliations:** 1 Department of Fixed Prosthodontics, Faculty of Dental Medicine, Damascus University, Damascus, SYR

**Keywords:** lava plus, e.max, high-translucency zirconia, translucency, cad/cam, full ceramic crowns

## Abstract

Introduction

Nowadays, cosmetic demands are the first reason to visit dental clinic. However, most ceramic prostheses need an excessive removal of the dental structure, whether for full crowns or veneer preparation. With the innovation in ceramic materials, high translucent zirconia that demands minimal preparation with high aesthetic results was used for computer-aided design/computer-aided manufacturing (CAD/CAM) Full ceramic crown fabrication. The objective of this study was to compare the translucency properties of CAD/CAM full ceramic crowns fabricated from glass ceramics (E.max) and high translucency zirconia (Lava plus, 3M EPSE).

Material and methods

The sample consisted of 60 full ceramic crowns divided into two groups; the first group consisted of 30 IPS E.max CAD/CAM full ceramic crowns, while the second one consisted of 30 high translucent zirconia CAD/CAM full ceramic crowns. Translucency for both groups was evaluated directly after cementation by observing the blue light passing through the prostheses. Pearson Chi-Square test was used to study the difference in translucency between the two study groups.

Results

IPS E.max CAD showed a higher translucency compared to lava plus high translucency zirconia; in addition, this difference was statistically significant (p=0.028).

Conclusion

Within the limitation of this study, we found that the translucency of dental ceramic was affected by the ceramic material. As IPS E.max CAD was significantly higher in terms of translucency compared to High Translucent Zirconia.

## Introduction

Nowadays, cosmetic demands are the first reason to visit a dental clinic, and full ceramic prostheses usually meet these demands [1]. Dental porcelain is an inorganic compound consisting of oxygen and one or more metals or semi-metals such as aluminum, lithium, selenium, calcium, sodium, titanium, and zirconium [2]. It is a chemical inhibitor material with high biocompatibility. However, it is a brittle material that breaks when bent or when its temperature rises and falls quickly. It is characterized by high optical properties and the possibility of manufacturing it in different structures [2].

Feldspathic ceramic was used as a material for manufacturing fixed prostheses for long periods due to its high aesthetic properties and results [3]. Nevertheless, the constant developments in dental materials, especially for dental porcelain, led to several types of ceramics with different mechanical and aesthetic properties. E.max (IPS E.max, Ivoclar Vivadent, Germany) was one of the most used types, consisting mainly of glass ceramic reinforced with disilicate lithium crystals, which gives it bending resistance properties and excellent aesthetic properties [4]. Lithium disilicate (IPS e.max) contains a high percentage of crystalline (50% or higher), which improves mechanical strength and gives it high aesthetic properties with a natural appearance [5]. Clinical success and durability are related to the amount of remaining enamel after preparation. However, adhering to resin cement increases its fracture and pressure resistance [6].

Recently, new aesthetic porcelain materials have been used, such as high translucent zirconia (3M™ Lava™ Plus High Translucency Zirconia, United States), which is a versatile material and can be utilized for single anterior and posterior restorations, for bridges up to three units, and for implant crowns, and characteristic by high biocompatibility and high aesthetic properties [7]. Its translucency is determined by the density and grain size of the sintered material, the presence of impurities and structural defects that lead to light absorption and scattering that can reduce translucency, as well as lower alumina content and regular distribution maintain aging stability and increases translucency [7].

High translucent zirconia can afford 1,500n of fracture forces with a preparation thickness of 0.6 mm, while lithium di silicate needs a preparation thickness of 1.2 mm to afford the same forces. Thus, High translucent zirconia is considered a conservative prosthesis that needs minimal preparation [8].

Fabricating prostheses by Computer-aided design/computer-aided manufacturing (CAD/CAM) techniques have many advantages over traditional laboratory procedures, as it is faster, easier to use, and saves more time by skipping waxing, casting, pouring, and firing procedures. In addition, CAD/CAM systems reduce technical problems and produce a precise prosthesis with a lower possibility of fracture, which refers to the absence of pores in its inner surfaces [9].

Several studies have evaluated the translucency properties of E.max full ceramic crowns fabricated using CAD/CAM techniques. However, minimal studies have mentioned Lava plus high translucent zirconia crowns, therefore, this study aimed to compare the translucency properties of CAD/CAM full ceramic crowns fabricated from glass ceramics and high translucency zirconia.

## Materials and methods

Sample description

The study sample consisted of 60 CAD/CAM full ceramic crowns divided into two groups: the first group consisted of 30 CAD/CAM full ceramic crowns fabricated from glass ceramic (IPS e.max). Similarly, the second group consisted of 30 CAD/CAM full ceramic crowns fabricated from high translucent zirconia.

G-Power software version 3.1.9.4 (Heinrich Heine University Düsseldorf, Düsseldorf, Germany) was used to calculate the sample size. A significance level of 0.05 and a power of 95% were determined. As a result, a sample size of 30 patients was required for each group.

Patient recruitment

Crowns were performed for patients attending the Department of Fixed Prosthodontics of Damascus University Dental School who suffer from aesthetic problems in their teeth in the anterior and premolars regions.

The inclusion criteria were as follows: patient's age ranged from 18 to 50 years old, an obvious indication for full ceramic crowns (large restorations or caries, misalignment of the teeth, small interdental spaces after orthodontic treatment, teeth shape and size modification, stained teeth, crowding or titled teeth, congenital large enamel defaults that are causing an esthetic problem) in addition, absence of gingival and the periodontal inflammatory, normal or near normal anterior occlusion bite, absence of non-functional habit and good oral hygiene.

The exclusion criteria consisted of having a big modification on the occlusion bite, loss of more than two-thirds of the dental walls, i.e., the need to place a metal post and core under the final crown, and the loss of alveolar bone support for the root.

The main complaint and the patient medical and dental history were taken. Patients were questioned about their medical history and medications. Patients underwent intraoral and extraoral examinations. In addition, intra-oral pictures and pictures of the smiling status were taken. Furthermore, each patient was asked about the expected aesthetic result. This information was filled in a specific form created for this study. Planmeca Romexis® Smile Design software was used to show the final results to the patients. Finally, patients' consent was obtained.

Teeth preparation and prosthetic stage

Teeth were prepared to receive full ceramic crowns as follows: for E.max crowns, guidance grooves with a depth of 1mm were made by depth cutting bur (DentalMart E-Commerce Pvt. Ltd), then flat end cylinder bur was used to complete the occlusal surface preparation; for axial walls preparation, black round end taper bur (1mm) was used. Finally, a red round-end taper bur (1mm) was used to determine the shoulder finish line. As well as the same steps were done for zirconia crowns. However, the preparation thickness ranged between 0.6 and 1mm for all surfaces, with 0.5mm thickness of the semi-shoulder finish line.

The gingiva was retracted using the appropriate size of the gingival retraction cords according to the gingival biotype (thick/thin) of each patient in order to determine the finish line area. After that, final impressions were taken using additional silicone (PRESIDENT The Original, Colten, Switzerland). On the other hand, alginate was used to take the impression of the opposite arch. The occlusal bite was registered by the wax bite. The impression was poured using gypsum type IV (Maruvest Speed, Megadental, Büdingen, Germany).

At the end of this session, a suitable shade was chosen by the dentist using Vita 3D shade guide. That shade was documented after getting the patients' approval; then temporary prostheses were made using intra-oral acrylic (R-Dental).

Fabrication of the full ceramic crowns using the CAD/CAM technique

The first step was applying a powder spray on the surfaces of the dies for the scanning process, which was performed using an extraoral scanner (T300, Medit, Korea), a “3D in Lab” designing program, and a computer. All of these subjects were connected to the laser scanner device (CCD), and after that, a 3D picture of the cast model was obtained on the computer screen.

After determining the finish line at the digital virtual model with a 40-micron virtual luting cement space and finishing the shape designing process, the milling process was divided into two stages. Stage one included milling the IPS E.max block using a “wet 360c” milling device (Imes-core company), while stage two included milling the Lava plus discs using a “Dry 250i” device (Imes-core company).

After completing the milling process, we obtained a zirconia disc containing the milled prostheses connected by zirconia wedges, and the same applies to the prostheses made of lithium disilicate, each crown was then separated using specific separating disks, whereas the crowns were unhardened completely and have a blue color shade.

A hardening oven (Programt P500) was used to start the crystallization process for E.max crowns, with the heating degree up gradually to prevent any following shrinkages. When the hardening step was done, glass ceramic (IPS e.maxceram) was applied over all crown surfaces.

Zirconia crowns were placed in a special crucible filled with zirconia granules to support them during their shrinkage resulting from condensation; after that, they were inserted into the condensing oven and remained for 9 hours and 20 minutes, the oven temperature was raised by 8 degrees per minute with stability for half an hour until it reached 900°, then it gradually raised again at a rate of 3 degrees per minute until it reaches 1,530° and remains constant for two hours at this temperature. At the end of the sintering process, it returns and decreases to room temperature. Sandblasting was carried out on the inner surface of these crowns, using aluminum oxide granules of size 110 microns and a pressure of 2 bar for 30 seconds, taking care not to approach the edges.

The final step for both groups was glazing, which was carried out using a glazing kit from Dental Direct company, the material was applied using a special brush, then the prostheses were placed in the thermal oven for 30 minutes at a temperature of 920°.

Final cementation

Before final cementation, provisional prostheses were removed, and full ceramic crowns were trying-in by examining the internal fit, interproximal points, high occlusal points (if any), and color matching with adjunct teeth and prosthesis. Furthermore, patients' approval of the aesthetic results was taken.

For IPS E.max crowns, etching with hydrofluoric acid (9%) was applied for 90 seconds, then washed and dried, double bonding agent was applied (MonoBond S) for 60 seconds and extended by airflow only.

For Lava plus high translucent zirconia crowns, sandblasting for the inner surfaces was done using a Pencil sanding tool for one minute, after which it was washed with running water for one minute, then primer containing MDP particulars (Bisico) was applied for 30 seconds according to the manufacturing company instructions.

In order to control saliva and ensure the complete dryness of the workspace, isolation was done using cotton rolls and a high-pressure suction. All surfaces of the prepared teeth were etched using phosphoric acid (37%) for 30 seconds, washed, and dried, then the chalky appearance was seen. The next step was to apply the dentin bonding agent (Tetric-N Bond) and spread it by a slight airflow only; light curing resin cement (Variolink N) was also put and spread on the whole inner surface of the crowns; after that, crowns were placed above the abutment surfaces.

Resin cement was light cured for 3-4 seconds only to easily remove the excess cement with dental floss for the proximal surfaces and by a dental probe and surgical blade type 12 for the buccal and palatal surfaces.

**Figure 1:**
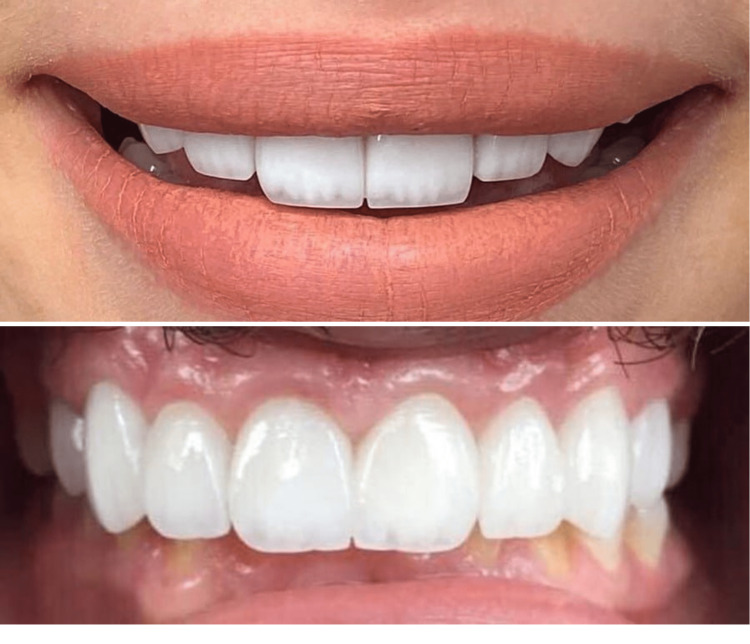
E.max CAD crowns after the final cementation

**Figure 2:**
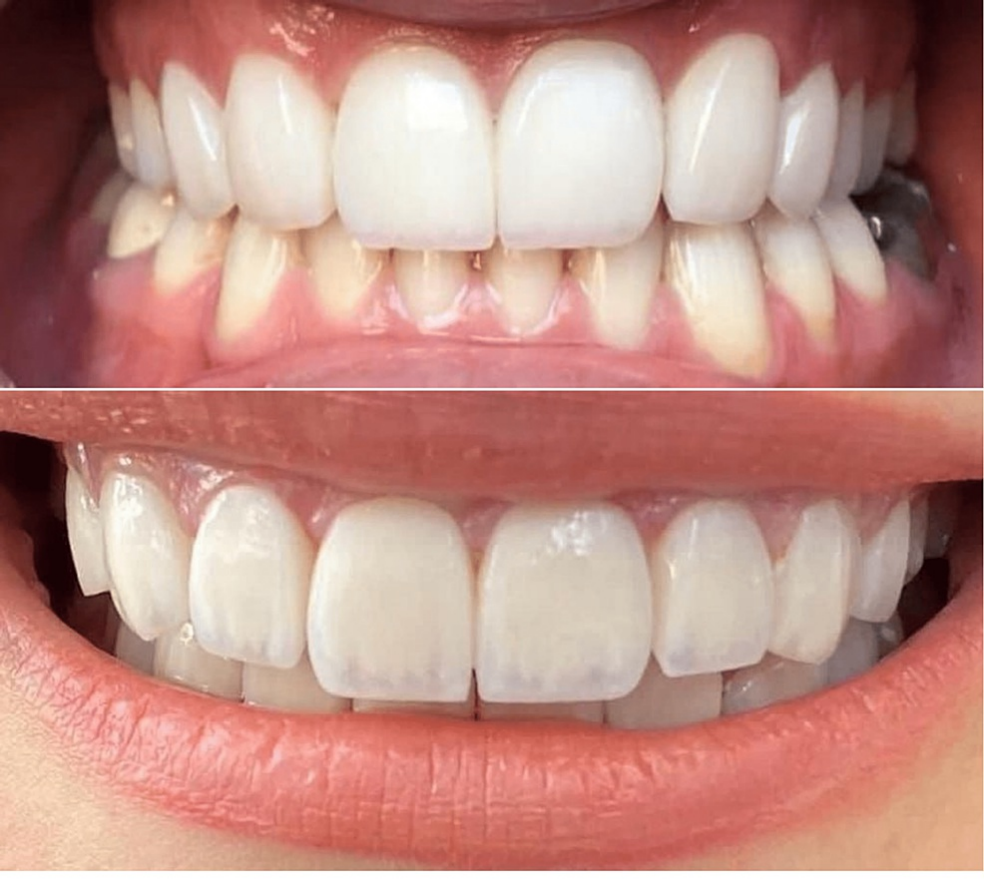
High-translucency zirconia crowns after the final cementation

Full curing was done by applying the light curing device for 60 seconds on each side. Finally, margins were finished using extremely soft diamond finishing burs and soft abrasive strips for the proximal areas (Figures 1, 2).

Translucency assessment

Translucency was assessed by applying the blue light emission by dental light curing unit, from the palatal surface of the measured tooth to evaluate the prosthesis translucency as if the light is visible from the buccal surface, the prosthesis is considered to be translucent, while if the light is not visible, the prosthesis is considered to be slightly translucent or opaque according to the amount of the passing light. The evaluation was done directly after cementation. 

Statistical analysis

Data were collected and exported to Microsoft Excel 2013 (Microsoft Corporation). Then, statistical tests were conducted using Statistical Package for the Social Sciences (SPSS) version 26 (IBM SPSS Statistics, Armonk, NY, USA), with a significance level of 0.05. Pearson's Chi-Square test was used to study the difference in translucency between the two groups.

## Results

Sample description

The sample consisted of 15 patients (five males, 10 females). Patients' ages ranged from 24 to 45 years, with an arithmetic mean of 35.9 years and a standard deviation of 6.1 years (Table 1). Five patients were smokers, and ten of them were non-smokers.

**Table 1:** Descriptive statistic of the sample age Min=minimum. Max=maximum.

	Arithmetic mean	standard deviation	Min	Max
Age	35.9	6.1	24.0	45.0

The distribution of teeth in the lava group was as follows: 10 central incisors, 10 lateral incisors, six canines, and four first premolars, while in the E.max group, the distribution was: 10 central incisors, 10 lateral incisors, four canines, four first premolars and two second premolar (Table 2).

**Table 2:** Frequencies and percentages of the prepared teeth

The tooth	E.max		Lava	
	Frequency	%	Frequency	%
Central incisor	10	33.3%	10	33.3%
Lateral incisor	10	33.3%	10	33.3%
Canine	4	13.3%	6	20%
First premolar	4	13.3%	4	13.3%
Second premolar	2	6.6%	-	0%

Results of translucency assessment

**Figure 3:**
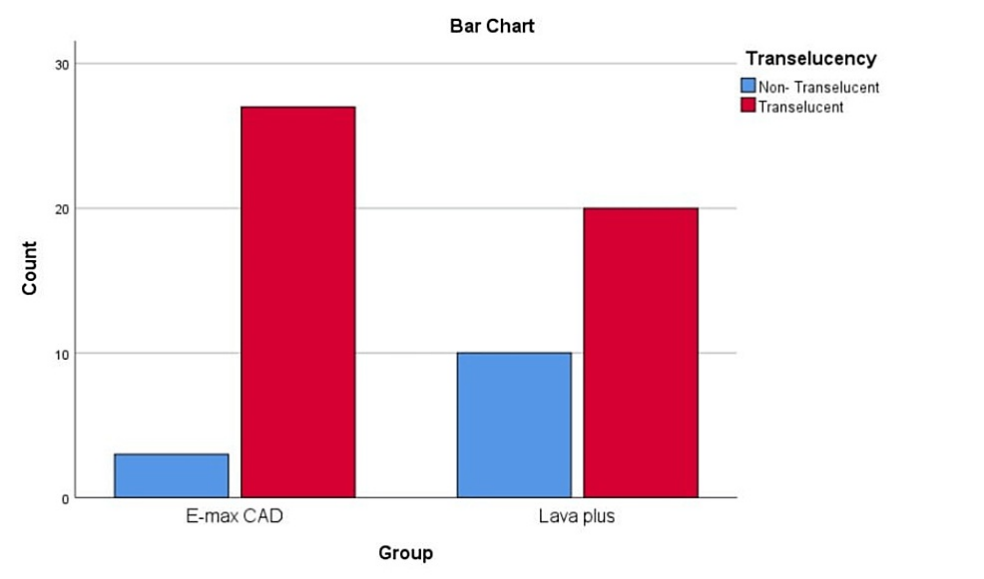
Bar chart of translucency percentage within both groups

After the end of cementation session, translucency was assessed, and gave the following results (Table 3, Figure 3).

**Table 3:** Group * Translucency Crosstabulation

			Translucency		Total
			Non- Translucent	Translucent	
Group	E-max CAD	Count	3	27	30
	Lava plus	Count	10	20	30
Total		Count	13	47	60

As a result, there was a difference in transparency between the two groups; thus, Pearson Chi-Square was used to detect whether or not this difference was statistical significance (Table 4).

**Table 4:** Chi-Square Tests df=degree of freedom.

	Value	df	Asymptotic Significance (2-sided)
Pearson Chi-Square	4.812^a^	1	.028

Therefore, IPS Emax CAD showed a higher translucency than high-translucency zirconia, with a statically significance difference (p=0.028).

## Discussion

Several materials were used to achieve the best esthetic results with different types of prostheses as fixed bridges, full crowns, and veneers. Thus, with the development of glass ceramics, some materials became more popular, such as lithium disilicate [10]. However, this material needs excessive removal from the dental structure, which causes some complication as pulpitis, hypersensitivity, and loss of prosthetic retention [11]. Recently, a new generation of translucent zirconia has appeared, such as Lava plus (3M EPS) and Cubic x2 (Dental Direkt), which need a preserve and minimal preparation as they need to remove half of the amount of dental structures removed when using other materials while achieving aesthetic and functional results that are close to or superior to them [12]. In addition, conservative preparation significantly affects the prosthetic durability and preservation of the pulp [13, 14]. So, this study aimed to compare the translucency properties of CAD/CAM full ceramic crowns fabricated from glass ceramics (E.max) and high translucency zirconia (Lava plus, 3M EPSE).

The sample consisted of 15 patients who attended the Department of Fixed Prosthodontics of Damascus University Dental School and suffered from aesthetic problems in their teeth appearances in the interior and premolars region% 66.7 of the patients were female, and generally, they are the most interested in having an esthetic appearance. In a cross-sectional study conducted at the Lithuanian University of Health Sciences (LSMU), it was reported that females were the most interested in smile shape and appearance and most demanding the esthetic aspects [15].

Translucency can be described as the quality of light passing through a material; thus, translucent material is made up of components with different indices of refraction. When light encounters a material, it can interact with it in several different ways [16]. These interactions depend on the wavelength of the light and the nature of the material [16]. Therefore, an LED dental light curing unit (3TECH LED-1007 - curing light - dental sky - USA) from which it is emitted blue light (light intensity: 5W > 1,100 mW/cm², full power mode, 485 nm wavelength) was used to assess translucency by applying the light from the palatal surface of the measured tooth to evaluate the prosthesis translucency, as if the light is visible from the buccal surface, the prosthesis is considered to be translucent, and if the light is not visible the prosthesis is considered to be slightly translucent or opaque according to the amount of the passing light, the evaluation was done directly after cementation.

In this study, IPS E.max showed a higher translucency compared to lava plus high translucency zirconia; in addition, this difference was statistically significant. The different translucency shown in both groups may be due to the different shade colors of the cemented prosthesis, as the shade color was chosen for each patient according to the color of his natural teeth. In addition, the buccal-lingual distance differs between teeth as well as between patients with the same tooth, which affects the distance traveled by the light.

The results of our study agreed with Church's study, which reported a higher translucency for E.max compared to different types of zirconia with different thicknesses. In addition, the translucencies of the zirconia materials were similar at each thickness [17]. As well as another study stated that CAD/CAM IPS E.max showed a higher translucency compared to zirconia ceramics and heat-pressing IPS Emax. However, high-translucent zirconia was the least translucent [18]. Sravanthi's study confirms the previous results, as it showed then even E.max press was higher in translucency compared to high translucent zirconia, as zirconia has got poor light transmission and high reflectance whereas lithium disilicate has got good light transmission [19].

High translucency zirconia is a tetragonal polycrystalline zirconia partially stabilized with 3mol-% Yttria engineered for high translucency and utmost strength. It has a lower Alumina content of 0.1%, optimally distributed within the material for maintaining aging stability, whereas Lithium disilicate (LS2) is classified as a glass-ceramic, in the class of particle-filled glass materials [20].

On the other hand, our results disagreed with Baldissara's study, which showed significantly higher translucency by using multilayered high translucent zirconia compared to IPS E.max [21]. This study had some limitations such as the size of the sample, the contents of the oral cavity of the lips and cheeks, which may affect the amount of reflected light, and not adding pressed glass ceramics (E.max) to the study groups. 

## Conclusions

Within the limitation of this study, we found that the translucency of dental ceramic was affected by the ceramic material as IPS E.max CAD was significantly higher in terms of translucency compared to High Translucent Zirconia when using it for interior and posterior restorations. This means that even with different ceramic thicknesses, IPS E.max showed higher translucency propriety.

## References

[1] Calamia J, Wolff M, Simonsen RJ (2007). Successful esthetics and cosmetic dentistry for modern dental practice.

[2] Anusavice KJ, Phillips RW (2003). Phillips’ science of dental materials. StLouis WB Saunders.

[3] Barizon KT, Bergeron C, Vargas MA, Qian F, Cobb DS, Gratton DG, Geraldeli S (2014). Ceramic materials for porcelain veneers: part II. Effect of material, shade, and thickness on translucency. J Prosthet Dent.

[4] da Cunha LF, Pedroche LO, Gonzaga CC, Furuse AY (2014). Esthetic, occlusal, and periodontal rehabilitation of anterior teeth with minimum thickness porcelain laminate veneers. J Prosthet Dent.

[5] Fasbinder DJ (2010). The CEREC system: 25 years of chairside CAD/CAM dentistry. J Am Dent Assoc.

[6] Friedman MJ (1998). A 15-year review of porcelain veneer failure--a clinician’s observations. Compend Contin Educ Dent.

[7] Schechner G, Dittmann R, Fischer A, Hauptmann H (2012). Contrast Ratios of Uncolored and Colored Zirconia Materials. https://www.researchgate.net/publication/266804746_Contrast_Ratios_of_Uncolored_and_Colored_Zirconia_Materials.

[8] Lughi V, Sergo V (2010). Low temperature degradation -aging- of zirconia: a critical review of the relevant aspects in dentistry. Dent Mater.

[9] Davidowitz G, Kotick PG (2011). The use of CAD/CAM in dentistry. Dent Clin North Am.

[10] Anusavice KJ, Shen C, Rawls HR (2012). Phillips’ science of dental materials. https://books.google.com/books?hl=en&lr=&id=gzUeKDhP-KQC&oi=fnd&pg=PP1&dq=Anusavice+KJ,+Shen+C,+Rawls+HR,+Phillips%E2%80%99+science+of+dental+materials.+Elsevier+Health+Sciences,+2012.&ots=BhRYs1JPqY&sig=Kg3L5y2OXo6WcWuZEo6iVarW-UU#v=onepage&q=Anusavice%20KJ%2C%20Shen%20C%2C%20Rawls%20HR%2C%20Phillips%E2%80%99%20science%20of%20dental%20materials.%20Elsevier%20Health%20Sciences%2C%202012.&f=false.

[11] Almutairi W, Aminoshariae A, Williams K, Mickel A (2021). The validity of pulp tests on crowned teeth: a clinical study. Eur Endod J.

[12] Kwon SJ, Lawson NC, McLaren EE, Nejat AH, Burgess JO (2018). Comparison of the mechanical properties of translucent zirconia and lithium disilicate. J Prosthet Dent.

[13] Winkelmeyer C, Wolfart S, Marotti J (2016). Analysis of tooth preparations for zirconia-based crowns and fixed dental prostheses using stereolithography data sets. J Prosthet Dent.

[14] Osborne JW (2020). Conservative tooth preparation, minimal intervention, and/or narrow-parallel preparations: a narrative. Oper Dent.

[15] Armalaite J, Jarutiene M, Vasiliauskas A, Sidlauskas A, Svalkauskiene V, Sidlauskas M, Skarbalius G (2018). Smile aesthetics as perceived by dental students: a cross-sectional study. BMC Oral Health.

[16] Muller H (2020). Concrete with improved visibility in low light conditions. Stellenbosch Univ.

[17] Church TD, Jessup JP, Guillory VL, Vandewalle KS (2017). Translucency and strength of high-translucency monolithic zirconium oxide materials. Gen Dent.

[18] Wang F, Takahashi H, Iwasaki N (2013). Translucency of dental ceramics with different thicknesses. J Prosthet Dent.

[19] Sravanthi Y, Ramani YV, Rathod AM, Ram SM, Turakhia H (2015). The comparative evaluation of the translucency of crowns fabricated with three different all-ceramic materials: an in vitro study. J Clin Diagn Res.

[20] Albakry M, Guazzato M, Swain MV (2004). Influence of hot pressing on the microstructure and fracture toughness of two pressable dental glass-ceramics. J Biomed Mater Res B Appl Biomater.

[21] Baldissara P, Wandscher VF, Marchionatti AM, Parisi C, Monaco C, Ciocca L (2018). Translucency of IPS e.max and cubic zirconia monolithic crowns. J Prosthet Dent.

